# Local alignment of generalized *k*-base encoded DNA sequence

**DOI:** 10.1186/1471-2105-11-347

**Published:** 2010-06-24

**Authors:** Nils Homer, Stanley F Nelson, Barry Merriman

**Affiliations:** 1 Department of Computer Science, University of California Los Angeles, Los Angeles, California 90095, USA; 2 Department of Human Genetics, David Geffen School of Medicine, University of California Los Angeles, Los Angeles, California, 90095, USA

## Abstract

**Background:**

DNA sequence comparison is a well-studied problem, in which two DNA sequences are compared using a weighted edit distance. Recent DNA sequencing technologies however observe an encoded form of the sequence, rather than each DNA base individually. The encoded DNA sequence may contain technical errors, and therefore encoded sequencing errors must be incorporated when comparing an encoded DNA sequence to a reference DNA sequence.

**Results:**

Although two-base encoding is currently used in practice, many other encoding schemes are possible, whereby two ore more bases are encoded at a time. A generalized *k*-base encoding scheme is presented, whereby feasible higher order encodings are better able to differentiate errors in the encoded sequence from true DNA sequence variants. A generalized version of the previous two-base encoding DNA sequence comparison algorithm is used to compare a *k*-base encoded sequence to a DNA reference sequence. Finally, simulations are performed to evaluate the power, the false positive and false negative SNP discovery rates, and the performance time of *k*-base encoding compared to previous methods as well as to the standard DNA sequence comparison algorithm.

**Conclusions:**

The novel generalized *k*-base encoding scheme and resulting local alignment algorithm permits the development of higher fidelity ligation-based next generation sequencing technology. This bioinformatic solution affords greater robustness to errors, as well as lower false SNP discovery rates, only at the cost of computational time.

## Background

DNA sequence comparison is a well studied problem in biology and bioinformatics [1-4]. Recently, a new DNA sequencing technology (ABI SOLiD sequencing) has been developed which does not measure each base directly, but instead measures DNA bases in pairs in an encoded form [5-7]. This technology has the potential to have greater error tolerance by differentiating biological variants from sequencing errors. In this manner, Homer et al. (2009) previously developed an algorithm to compare an encoded DNA sequence to a target reference [8], with a similar method independently derived in Rumble et al. (2009) [9]. These two algorithms do not significantly differ and therefore the proposed algorithm is compared to the algorithm presented in Homer et al. (2009). This two-base encoding and resulting local DNA sequence comparison algorithm can be utilized with global search strategies [9-12] for whole-genome sequencing with next-generation sequencing technology [13].

The central advantage of the two-base encoding scheme is that the false discovery rate of a single nucleotide polymorphism (SNP) is reduced, since two specific adjacent errors are required to produce a SNP call. In fact, only one-fourth of all adjacent errors would result in a false call. This significantly reduces the probability of falsely observing a SNP, with current machines exhibiting a color read error rate less than 5%. Nevertheless, the currently implemented two-base encoding is not the only possible encoding. Therefore a generalized *k*-base encoding scheme is presented, whereby *k *consecutive bases are simultaneously observed. The algorithm of Homer et al. (2009) is extended to solve the DNA sequence comparison problem of comparing a *k*-base encoded DNA sequence and a target reference DNA sequence. Intuitively, with greater *k*, the number of errors required to falsely discover a SNP also becomes greater, thus allowing machine errors to be accurately identified, and even corrected, while retaining sensitivity to detect real base changes. Simulations are performed to explore the improved power of higher order *k*-base encoding schemes, as well as the performance time when utilizing these encodings. These simulations explore the case for adapting *k*-base encoding schemes in ligation-based next generation sequencing technology.

## Results and Discussion

Simulations were performed to explore the power and performance of *k*-base encoding as well as the *k*-base encoding local alignment algorithm (see Methods). Reads were simulated using sequences both with a uniform error-rate, as well as using sequences with an error-rate modeled after real-world data. In this discussion,"1-base encoding" and "no encoding" are used interchangeably. The power to align a sequence with or without SNPs is defined as the fraction of reads where the read sequence is aligned to the reference with the same alignment score as if the sequence were aligned to the correct location (see Methods). The fraction of reads where the read is aligned to call a SNP, and where the original sequence had no SNP, defines the false positive SNP discovery rate. Similarly, the fraction of reads where the read is aligned not to call a SNP, and where the original sequence had a SNP, defines the false negative SNP discovery rate.

**Figure 1:**
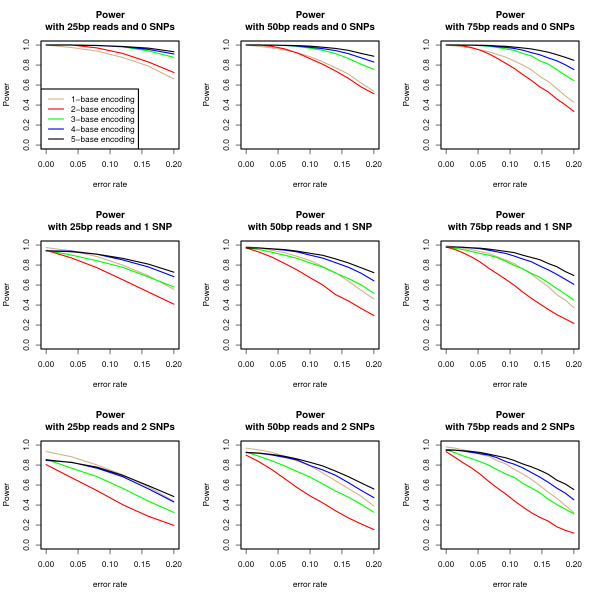
**Power of *k*-base encoding**. Power calculated as the fraction of reads that correctly align. 10, 000 simulated reads from the E. Coli genome were generated.

Plotted in Figure 1 is the power to align encoded sequences of varying length (25, 50, and 75 bp) with 0-2 SNPs or base substitutions given a fixed uniform error rate for *k *= 1...5 (see Methods). The power decreases similarly for all read lengths as the error-rate increases given a fixed number of SNPs. Furthermore, the power decreases substantially when the number of SNPs in the sequence is increased at a fixed error-rate and read length. The power of 1-base encoding, observing each base directly, does not diminish as much as *k*-base encoded sequences (*k *> 1) when more SNPs are introduced. This is due to SNPs and observational errors being equivalent in the 1-base encoding case. In these simulations, *k*-base encoding is more powerful when *k *> 2 for 0 SNPs, when *k *> 3 for 1 SNP, and when *k *> 4 for 2 SNPs and longer read lengths. It is important to note that in many cases when the alignment score between the best alignment and correct alignment differ, the decoded base sequences match. Therefore, the false positive and false negative SNP discovery rates are examined later.

To assess the power of *k*-base encoding utilizing real-world error rates, the color error (encoding error) rates were estimated from a previous run of an ABI SOLiD v2 sequencer (see Methods). The power of aligning such sequences was assessed in the presence of 0-2 SNPs for *k *= 1...5 (Table 1). For sequences with no SNPs, the power of k-base encoding increases as *k *increases. However in the context of an increasing number of SNPs and for *k *> 1 the power of *k*-base encoding is more ambiguous. For one SNP, *k *= 2 performs more poorly than no encoding, while *k *> 2 improves on the lower width encodings. For two SNPs, both *k *= 2 and *k *= 3 perform more poorly than no encoding, with only *k *> 3 having better power than no encoding. Thus, error correction is increased with greater *k *when no SNPs are present. However, if the goal is to find variants, a large enough *k *must chosen carefully to justify the penalty in performance.

**Table 1:** Power of *k*-base encoding assuming a real-world per-base error-rate

*k *(encoding width)	Power (0 SNPs)	Power (1 SNP)	Power (2 SNPs)
1	0.877	0.847	0.820

2	0.931	0.824	0.706

3	0.963	0.876	0.784

4	0.964	0.911	0.834

5	0.965	0.911	0.840

Power calculated as the fraction of reads that correctly align. 10, 000 simulated 50 bp reads from the E. Coli genome were generated with an estimated real-world error rate.

**Figure 2:**
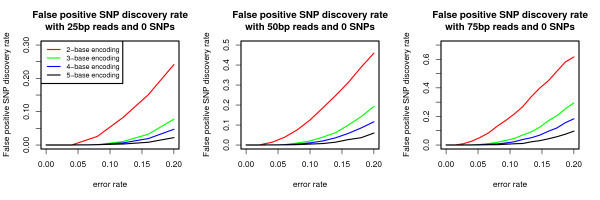
**False SNP discovery rate for *k*-base encoding**. False positive SNP discovery rate calculated as the fraction of reads that have a SNP call after alignment when no SNP call is expected. 10, 000 simulated reads from the E. Coli genome were generated.

**Figure 3:**
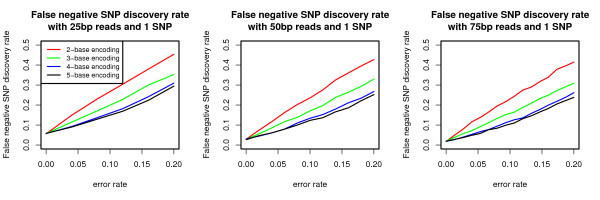
**False negative SNP discovery rate for *k*-base encoding**. False negative SNP discovery rate calculated as the fraction of reads that do not call a SNP after alignment when a SNP call is expected. 10, 000 simulated reads from the E. Coli genome were generated.

The false positive SNP discovery rate is evaluated for 25, 50, and 75 base-pair reads (Figure 2). With no encoding, SNPs and errors are not distinguishable, and therefore *k *= 1 is omitted from this discussion. As expected, the false positive SNP discovery rate decreases as *k *increases. Nevertheless, only above a five percent error rate does 2-base encoding begin to find false SNPs, and at approximately ten percent error rate do all encodings considered begin to falsely discover SNPs. Assessed in Figure 3 is the false negative SNP discovery rate. Similarly to Figure 2, the false positive SNP discovery rate, the false negative SNP discovery rate decreases as *k *increases. For low error rates, both of the above metrics are either zero (for Figure 2) or are less than 20% (for Figure 3). Thus, the settings can be interpreted as being conservative, sacrificing power to find true SNPs for decreasing the false positive SNP discovery rate.

**Figure 4:**
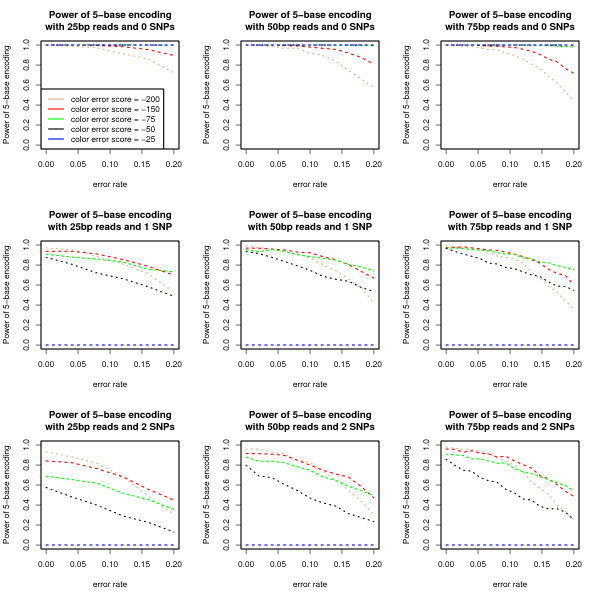
**Flexibility of scoring systems for 5-base encoding**. Power of scoring system evaluation for 5 base encoding. 1, 000 simulated reads from the E. Coli genome were generated.

To illustrate the flexibility of *k*-base encoding to be tuned for specific scenarios, the power of 5 base encoding is examined when the score for a color substitution is varied, and for 25, 50, and 75 base-pair reads with 0 - 2 SNPs. Intuitively, the various color substitution scores correspond to preferring a given number of color errors over a SNP and possibly fewer color errors (see Methods). As the color error score decreases, the encoding begins to prefer decoding with SNPs rather than with color errors. The various scoring schemes allow for a clear trade off in power to detect color errors over the power to detect SNPs (Figure 4). For example, the color substitution score of -25 allows the full correction of 50 bp reads with up to a 20% error rate. However, once a SNP is introduced it has zero power. Alter-natively, the color substitution score of -200 finds SNPs in the low error case, but with higher error data the power to detect only the given SNP(s) is confounded as more SNPs are falsely detected. With zero SNPS, color scores of -25, -50, and -75 have almost perfect power.

The performance time of *k*-base encoding scales exponentially with increasing *k *(see Methods). This is confirmed by plotting the timing information from the previous uniform error-rate simulations (Table 2). Since the optimizations found in Homer et al. (2009) [10] are not pursued, increased variability was observed when the number of SNPs increased. However, an exponential increase in running time occurs as the encoding width (*k*) increases. This has practical implications, whereby producing empirical data that is *k*-base encoded is possible, but is computationally infeasible to decode as the number of short-reads is in the millions, if not billions for typical experiments.

**Table 2:** Performance of *k*-base encoding

*k *(encoding width)	Time in s (0 SNPs)	Time in s (1 SNP)	Time in s (2 SNPs)
1	7	7	7

2	65	65	65

3	403	346	403

4	2178	2166	2178

5	23464	23460	23466

Performance time (in seconds) of *k*-base encoding assuming a real-word per-base error-ate on 50 bp reads presented in Table 1.

Nevertheless, this exponential increase in running time could be significantly reduced at the cost of completeness by using methods initially adopted for protein similarity search and sequence comparison [14,15]. A global search strategy is employed to put constraints on the possible search paths thereby significantly reducing the search space. Typically these constraints force the alignment path to proceed along diagonals in the dynamic programming matrix, reducing the dimensionality of the problem. These constraints are specifically used in the implementation of BFAST for both 1-base and 2-base encoded data [10]. The positions where the read and reference match during the BFAST's global search strategy are annotated and aggregated such that in the local alignment the read and reference must match (i.e. constraining the solution path to pass through a diagonal). In BFAST's case, the global search strategy searches over an 2-base encoded reference for *k *= 2 constraining the color transitions. The constraints employed by BFAST could be applied for *k *greater than two, yielding significant but unknown speed improvement.

## Conclusions

The generalized *k*-base encoding scheme and resulting local alignment algorithm presented here have the ability to more powerfully differentiate between encoded sequencing errors and true DNA variants. These schemes can be used in practice to tolerate high error rates in the raw data. Alternatively, the per-base accuracy of sequencing can be improved. The goal in most sequencing projects is to sensitively and specifically detect variants. The technology and encoding scheme must not only have sufficient power to detect variants but to also not overwhelm the true variants with false variants. The results demonstrate that higher encoding schemes not only improve the power of detecting variants, but also significantly reduce the false positive SNP discovery rate. Having multiple observations of a specific variant or genomic co-ordinate (higher coverage) is in most cases able to overcome sequencing error. Nevertheless, these encoding schemes could allow low coverage data to accurately detect variants. Furthermore, for cancer specific studies, where the sample may be a heterogeneous population of cells, these encodings could reduce the minimum detection level (allele frequency) in the cancer cell population as the fewer observations can be more confidently trusted.

Currently a two-base encoding system is used by ABI SOLiD sequencing technology. Some other next-generation sequencing technologies could also adopt an encoding system to improve their performance and accuracy. Furthermore, algorithms that perform multiple sequence alignment or local reassembly could also utilize the power of the encoding scheme presented here. It is interesting to note that error correction utilizing encoded DNA sequence could be performed if single bases or sets of bases were observed more than once. Utilizing various encoding schemes, this error correction would necessarily not rely on a target DNA reference comparison, thereby eliminating the expensive exponential increase in time for higher order encodings (larger *k*). Future investigation of such pre-alignment error correction schemes and algorithms is intended.

## Methods

### Generalized *k*-base encoding

Given an *k*-base encoded DNA sequence *c *= *c*_1_, ..., *c*_*n*_, it is the goal of the proposed algorithm to minimize the edit distance between c and some regular DNA sequence *y *= *y*_1_, ..., *y*_*m *_given a set of valid edit operators Σ. The DNA alphabet is assumed to be Λ = {*A, C, G, T*} and the valid edit operators include a color substitution, base substitution, a deletion, and an insertion. Similar to Homer et al. (2009), the color substitution operator is required to be applied before applying the insertion, deletion, or base substitution operators. Each operator is assigned a score, with ∏(*C*_1_, *C*_2_) and Δ(*B*_1_, *B*_2_) corresponding to the color substitution scoring and base substitution scoring functions respectively. To model insertions and deletions, affine gap penalties are used whereby a score of *ρ *is applied for the first insertion (or deletion), with ϵ applied for any consecutive insertion (or deletion) that extends the insertion (or deletion). It is assumed that for any bases *B*_1 _≠ *B*_2 _and for any colors *C*_1 _≠ *C*_2 _that 0 ≤ Δ(*B*_1_, *B*_1_), Δ(*B*_1_, *B*_2_) < 0, 0 ≤ ∏(*C*_1_, *C*_1_), ∏(*C*_1_, *C*_2_) < 0, *ρ *< ϵ < 0. These scoring assumptions penalize edits that change the encoded or decoded DNA sequence relative to the reference, thereby ensuring their similarity.

To illustrate the encoding and decoding method used by this technology, let *x *= *x*_1_, ..., *x*_*n *_be a DNA sequence. To encode a DNA sequence, the function Φ^*k*^(*B*_1_, ..., *B*_*k*_) is defined to return a color *C*_*k *_using the bases *B*_1_, ..., *B*_*k*_, where B_*i*-1 _occurs before B_*i *_in the sequence. For example, to encode the DNA sequence *x *= *x*_1_, ..., *x*_*n*_, first a known start adaptor *p *= *p*_1_, ..., *p*_*k*-1 _∈ Λ^*k*-1 ^is assumed. Next, the function Φ^*k *^is iteratively applied to the concatenation of *p *and *x*. *c *In this case, *c*_1 _= Φ^*k*^(*p*_1_, ..., *p*_*k*-1_, *x*_1_), *c*_2 _= Φ^*k *^(*p*_2_, ..., *p*_*k*-1_, *x*_1_, *x*_2_), ..., *c*_*n *_= Φ^*k*^(*x*_*n*-*k*+1_, *x*_*n*_). The adaptor sequence p is known in practice and is used in the physical chemistry of the sequencer (for *k *= 2), not the DNA sequence in question [5-7].

The encoding function Φ^*k *^(*B*_1_, ..., *B*_*k*_) transforms each base *B*_*i *_into an integer representation (*i.e*. *A *= 0, *C *= 1, *G *= 2, *T *= 3), sums the integer values, and returns the result modulo four. Let *δ *return the integer representation of a base as described above, then Φ^*k*^(*B*_1_, ..., *B*_*k*_) = *mod *|Λ|. Modulo four is chosen since four colors are used in current technologies. The properties of the modulo-four-specific encoding are discussed after how a *k*-base encoded sequence is decoded.

To decode an encoded sequence, the function Γ^*k*^(*B*_1_, ..., *B*_*k*-1_, *C*) is defined to return the decoded base *B*_*k *_using the encoded color *C *and the previous bases *B*_1_, ...*B*_*k*-1_. To compute Γ^*k*^(*B*_1_, ..., *B*_*k*-1_, *C*), *B *must be solved for in the equation *mod *|Λ|, which is easily solved. For example, to decode the encoded sequence *c *= *c*_1_, ..., *c*_*n *_with a known start adaptor *p *= *p*_1_, ...*p*_*k*-1 _∈ Λ^*k*-1^, Γ^*k *^is iteratively used. The decoded sequence will be *x*_1 _= Γ^*k*^(*p*_1_, ..., *p*_*k*-1_, *c*_1_), *x*_2 _= Γ^*k*^(*p*_1_, ..., *p*_*k*-2_, *x*_1_, *c*_2_), ..., *x*_*n *_= Γ^*k*^(*x*_*n*-*k*+1_, *x*_*n*-1_, *c*_*n*_). Without the start adaptor *p*, there would be |Λ|^*k*-1 ^possible decodings of the encoded sequence.

This encoding function has two useful properties. First, if one base in x is changed to obtain a new DNA sequence *x'*, then the new *k *colors that encode the changed base in *x' *will differ from the corresponding *k *colors in *x*. For example, if *k *= 5 then changing one base in *x *to obtain new DNA sequence *x' *will cause there to be 5 color differences between the encoded version of x and the encoded version of *x'*. A second useful property is if one color in the encoded version of *x *is changed to obtain a new encoded version, say *c'*, then every base in the decoded version of *c' *that occurs after the changed color will be different from the corresponding bases in *x*. The first property defines the signature of base substitutions in the encoded sequence, which becomes pronounced as *k *increases. The second property tells us that an encoding error will modify all bases after the encoding error. Intuitively, one can simplify by observing that for any base substitution there exists a set of *k *consecutive errors that can be applied to achieve the same base substitution, and therefore if variants are being searched for (base substitutions in particular) then constraints should be placed on the scoring system to prefer calling base substitutions rather than color substitutions when comparing to a reference. An example of such a constraint is given that removes the above ambiguity. Suppose there exists a sub-sequence of the encoded read , such that they all encode a base . Next, consider the reference base *B*_*i *_≠  and the *k *"colors" that encode *B*_*i*_: *C*_*i*_, ..., *C*_*i*+*k*-1_. Let the *i*th DNA base be  such that  encode *B*_*i*_. The following constraint is made to prefer a base change and *k *color matches to a base match and *k *consecutive color mismatches:(1)

In this case, it is assumed that *C*_*j *_≠  and *B*_*i *_≠  (∀*i *≤ *j *≤ *i *+ *k *- 1). Numerous other constraints based on real-world requirements are possible but not explored here.

### The Algorithm

Suppose that a color sequence *c *= *c*_1_, ..., *c*_*n *_with a known adaptor *p *∈ Λ^*k*-1 ^is to be aligned to a reference sequence *y *= *y*_1_, ..., *y*_*m*_. To search over all possible base substitution, base insertion, base deletions, and color substitutions, define a recursive formula that is the repeated calculation in the dynamic programming algorithm.(2)

Intuitively, Equation 2 is filling in an *n *by *m *matrix, with each cell in the matrix containing 3 × Λ^*k*-1 ^sub-cells. It is interesting to observe for *k *> 2 when computing  and  that 2 considers only previous sub-cells that are consistent with the current sub-cell. In other words, the first *k *- 2 bases of *σ *(the current sub-cell) must correspond to the last *k *- 2 bases of *ϕ *(the previous sub-cell). In this formula, the *h *sub-cells represent bases present in *y *but not in *x*, while *v *sub-cells represent bases present in *x *but not in *y*. The *s *sub-cells represents a base *x*_*i *_aligning to a base *y*_*i *_in the reference sequence *y*.

An alignment that begins or ends with a deletion is ignored, since a sequence must span the break-point for the deletion to be observable (with respect to *x*). This is a valid assumption when *x *is the observed sequence, and *y *is a fixed reference. An insertion followed by a deletion (or vice versa) is ignored since this is rare for short DNA sequences, although to consider such an event would require minimal changes to the above formula.

If the color match scores are the same (∀*i *≠ *j*, ∏(*c*_*i*_, *c*_*i*_) = ∏(*c*_*j*_, *c*_*j*_)) and all color mismatch scores are the same (∀*i *≠ *j, k*, ≠ *l*, ∏(*c*_*i*_, *c*_*j*_) = ∏(*c*_*k*_, *c*_*l*_), then Equation 2 can be simplified. The recursive rule for the  term becomes:(3)

This modification forces any color substitution to be at the beginning or end of any inserted bases in *x *and can reduce the complexity of the algorithm dramatically. The intuition behind this simplification stems from not having any reference bases to which to compare the inserted bases. This forces the maximum score path through the insertion to have no color errors

Various initializations are possible, and the alignment of the entire encoded DNA sequence *x *to some subsequence of *y *is presented here. Therefore, the initialization becomes for ,  if *σ *= Γ^*k*^(*p*, *c*_*i*_) and  otherwise, and for  if *σ *= Γ^*k*^(*ϕ*, *c*_*i*_), so that the local alignment spans the entire encoded sequence and insertions are allowed at the beginning of any alignment. Notice that if there were any color errors within the beginning an insertion, they are aligned such that they occur at the end of the insertion.  for *j *≥ 0 is initialized so that the alignment does not begin with a deletion. The remaining initializations are:  for *j *≥ 0 *σ *= *p *and *σ *∈ Λ^*k*-1^, and  if *σ *= *p*,  otherwise, for *j *≥ 0 and *σ *∈ Λ^*k*-1^. These initializations enforce that the starting adaptor is *p*. To find the optimal local alignment, the cells  and  are searched over for a cell with maximum score, again ignoring the case where the alignment ends with a deletion. Backtracking is used to recover maximum scoring alignment.

This algorithm is in fact finding the shortest path on a graph with the nodes defined by the sub-cells of the matrix, and the edges weighted and defined by the recursive rules. To analyze the time complexity, it is observed that given the *k*-base encoding scheme for each sub-cell of type *h*, *v*, and *s *there are |Λ|^*k*-1 ^sub-cells. For each  sub-cell it is required to calculate the maximum over two values. For each  and  sub-cell it is required to calculate the maximum over 3 × |Λ| values. Therefore for each cell, various maximum must be computed over 2 × |Λ|^*k*-1 ^+ |Λ|^*k*-1 ^× 3 × |Λ| + |Λ|^*k*-1 ^× 3 × |Λ| = 2 × |Λ|^*k*-1^(1 + 3 × |Λ|). In practice, |Λ| = 4 and therefore various maxima must be computed over 26 × 4^*k*-1 ^values. From this analysis, it is clear that the running time of this algorithm is *O*(*nm*|Λ|^*k*^), which unfortunately scales exponentially with respect to the length of the encoding *k*.

### Simulations

Simulations were performed to assess the power and performance of *k*-base encoding, for *k *= 1...5. Sets of 10, 000 test sequences were randomly sampled from E. Coli (DH10B, NC_010473, CP000948). All sequences in a given set had a fixed read length (25, 50, 75), a fixed error-rate (0, 0.01, ..., 0.2), and a fixed number of SNPs (0, 1, 2). For the case of the 1-base encoding (*k *= 1), the standard Smith-Waterman algorithm was used, where errors were modeled as base changes, requiring that SNPs and base errors do not co-occur. For the case of *k*-base encoding with *k *> 1 errors were modeled as color substitutions (encoding errors). Similar to Homer et al. (2009), an alignment is defined to be accurate or correct if the returned alignment has the same score (or likelihood when the scores represent log- likelihoods) as the true alignment, which is known by the nature of these simulations.

To allow for insertions and deletions, the original sequence is used (before applying errors and variants) with an additional 10 bp before and after as the reference or target DNA sequence. In accordance with Equation 1, ϵ = - 50, *ρ *= - 175, ∏(*C*_1_, *C*_2_) = -125 (*C*_1 _≠ *C*_2_), ∏(*C*_1_, *C*_1_) = 0, Δ(*B*_1_, *B*_2_) = - 150(*B*_1 _≠ *B*_2_) and Δ(*B*_1_, *B*_1_) = 50. Due to these initializations, the optimization in Equation 3 is able to be performed. To model real-world error-rates, the simulated error-rates are learned from a run of an ABI SOLiD sequencer (50 base pairs), utilizing the aligned reads to calculate the 2-base encoding error, which is inherently dependent on the decoding algorithm used Homer et al. (2009). The error-rate was not uniform by sequencing position, therefore producing a color-error-rate for each position in the 50 color sequence reads. The observed error rate for each sequencing position was: 0.014, 0.005, 0.006, 0.007, 0.006, 0.006, 0.006, 0.008, 0.008, 0.008, 0.007, 0.006, 0.009, 0.009, 0.009, 0.009, 0.008, 0.015, 0.015, 0.012, 0.012, 0.011, 0.021, 0.021, 0.018, 0.019, 0.014, 0.037, 0.033, 0.031, 0.029, 0.022, 0.055, 0.052, 0.051, 0.043, 0.036, 0.087, 0.084, 0.076, 0.071, 0.060, 0.125, 0.118, 0.118, 0.108, 0.092, 0.179, 0.175, 0.184. To evaluate various scoring schemes of 5-base encodings, simulations of only 1, 000 test sequences were used due to running time limitations. For these evaluations a dual quad-core Intel Xeon E5420 machine at 2.5 GHz, with 32 GB of RAM and 2TB of RAID 0 disk space, was used, although the actual hardware requirements of the algorithm itself beyond CPU power are negligible relative to any modern computer.

### Scoring constraints 5-base encoding

Various scoring schemes were evaluated for 5-base encoding. For notational convenience, for all colors *C*_1 _≠ *C*_2 _and bases *B*_1 _≠ *B*_2 _let *CE *= ∏(*C*_1_, *C*_2_), *CM *= ∏(*C*_1_, *C*_1_), *BE *= Δ(*B*_1_, *B*_2_), and *BM *= Δ(*B*_1_, *B*_1_). Consider the scoring scenarios that satisfy one of the following constraints:

1. 5*CE + BM > 5CM + BE *(-*25*)

2. 5*CE + BM < 5CM + BE and 4CE + CM + BM > CE + 4CM + BE *(-*50*)

3. 4*CE + CM + BM < CE + 4CM + BE *and *3CE+2CM+BM > 2CE+3CM+BE *(-*75 *and -*150*)

4. 3*CE +2CM +BM < 2CE +3CM +BE *and *2CE+3CM+BM > 3CE+2CM+BE *(*200*)

5. 2*CE +3CM +BM < 3CE +2CM +BE *and *1CE + 4CM + BM > 4CE + CM + BE*

6. *CE + 4CM + BM < 4CE + CM + BE*

Intuitively, these scenarios try to decide if a given set of color errors should be preferred if they can be explained by a SNP and possibly other color errors. For example, the first scenario always prefers calling color errors over anything that can be explained by a SNP. The second scenario will prefer to explain the encoding with a SNP if it results in no color errors, but does not prefer to explain the encoding with a SNP if it is accompanied by any color errors. In the extreme, the last scenario would prefer to explain all color errors as a combination of a SNP and possibly color errors.

Nevertheless, given the assumptions that *BM *≥ 0, *CM *≥ 0, *BE *< 0, and *CE *< 0, it is observed that scenarios 5 - 6 are not possible. For example, constraint is equivalent to the following constraint: *CM + BM < CE + BE *and *4CE + BM > 3CE + BE*. Intuitively, our assumptions prefer to penalize color errors and nucleotide variants, rewarding color matches and nucleotide matches, thereby making the left-side of the above constraint without a solution. If scenarios 5-6 are truly desired, instead of considering color errors, color matches, base errors, and base matches separately, a joint function could be considered conditional on the various combinations of events over the *k *base and color window. This would also allow for the incorporation of any specific experimental bias for the combinations of events, or even specific bases.

In the above constraints color error scores are given that satisfy the constraints given the previously defined base match, base substitution, and color match scores. The score -150 is also included, which was previously used, to illustrate that there is flexibility even within these constraints to tune the scoring scheme.

## Authors' contributions

BM and NH conceived of *k*-base encoding. NH conceived of and implemented the algorithm, and performed the analyses. BM and SFN advised on the development and analysis of the method, and producing the manuscript. All authors read and approved the final manuscript.
